# Increased prescriptions for irritable bowel syndrome after the 2018 Japan Floods: a longitudinal analysis based on the Japanese National Database of Health Insurance Claims and Specific Health Checkups

**DOI:** 10.1186/s12876-022-02342-6

**Published:** 2022-05-26

**Authors:** Yuji Okazaki, Shuhei Yoshida, Saori Kashima, Daisuke Miyamori, Masatoshi Matsumoto

**Affiliations:** 1 Department of Emergency Medicine, Hiroshima City Hiroshima Citizens Hospital, 7-33 Motomachi, Naka-ku, Hiroshima City, Hiroshima 730-8518 Japan; 2 Department of Community-Based Medical Systems, Graduate School of Biomedical and Health Sciences, Hiroshima University, 1-2-3 Kasumi, Minami-ku, Hiroshima, 734-8551 Japan; 3 Environmental Health Sciences Laboratory, Graduate School of Advanced Science and Engineering, Hiroshima University, 1-5-1 Kagamiyama, Higashi-Hiroshima, Hiroshima 739-8529 Japan; 4 Department of General Internal Medicine, Hiroshima University, 1-2-3 Kasumi, Minami-ku, Hiroshima, 734-8551 Japan

**Keywords:** Irritable bowel syndrome, Post-infectious irritable bowel syndrome, Natural disaster, Mental stress, Epidemiology

## Abstract

**Background:**

The frequency and intensity of natural disasters are increasing worldwide, which makes our understanding of disaster-related diseases more important than ever. Natural disasters cause mental stress and infectious diarrhea, but the causal relationship between disasters and a potential consequence of these conditions, irritable bowel syndrome (IBS), is unreported. The 2018 Japan Floods, which took place in July 2018 was one of the largest water disasters in Japan’s recorded history. We investigate the change of drug prescriptions for IBS between disaster-suffers and non-sufferers throughout the disaster period to examine the relationship.

**Methods:**

This is a retrospective cohort study based on the Japanese National Database of Health Insurance Claims and Specific Health Checkups in flood-stricken areas between July 2017 and June 2019. We included subjects older than 15 years of age who had visited a medical institution or been hospitalized in the hardest-hit areas of the disaster. Ramosetron, polycarbophil calcium, and mepenzolate bromide (IBS drugs) approved solely for the treatment of IBS in Japan were analyzed. The monthly rate of prescriptions for IBS drugs was compared between municipality-certified disaster victims and non-victims using a controlled interrupted time series analysis. For those who were not prescribed IBS drugs before the disaster (non-users), the occurrence of an IBS drug prescription after the disaster was evaluated using a multivariable logistic regression analysis adjusted for gender and age.

**Results:**

Of 5,287,888 people enrolled, 32,499 (0.61%) were certified victims. The prescription rate for IBS drugs among victims increased significantly by 128% immediately after the disaster, while it was stable among non-victims. The trend for the post-disaster prescription rate among victims moved upward significantly when compared to non-victims (0.01% per month; 95% confidence interval (CI) 0.004–0.015; P = 0.001). Among non-users, the occurrence of an IBS drug prescription for victims was 0.71% and was significantly higher than non-victims (0.35%, adjusted odds ratio 2.05; 95% CI 1.81–2.32).

**Conclusions:**

The 2018 Japan Floods increased the rate of prescriptions for IBS drugs, suggesting that the disaster caused or worsened IBS among victims.

**Supplementary Information:**

The online version contains supplementary material available at 10.1186/s12876-022-02342-6.

## Background

Large-scale natural disasters can cause not only mental stress [1, 2], but also gastrointestinal diseases due to unsanitary living conditions, food and water [3]. In fact, gastroduodenal ulcers [4, 5] and acute infectious diarrhea [6, 7] reportedly increase in the acute phase of natural disasters. In Japan, large-scale earthquakes such as the Great East Japan Earthquake and the Kumamoto Earthquake have occurred successively in recent years, and torrential rains have also occurred frequently around the world [8]. It is thus increasingly important for clinicians to understand the relationship between natural disasters and gastrointestinal diseases.

Irritable bowel syndrome (IBS) is known to be caused by mental stress, changes in gut bacterial flora, and inflammation of the bowel mucosa [9, 10]. For example, post-traumatic stress disorder (PTSD) suffered early in life reportedly increases the risk of future IBS [11]. Post-infectious IBS is one of the types of this syndrome, which develops after acute infectious diarrhea [12]. It has been reported that post-infectious IBS develops in approximately 10% of patients with acute infectious diarrhea [13]. Both mental stress and bowel infection often take place among victims of natural disasters. Therefore, we can infer that natural disasters can induce new onset of IBS or exacerbate existing IBS symptoms among disaster sufferers. Studies in the literature testing this hypothesis, however, is scarce.

IBS has a significant impact on the daily life of patients, and the proper and well-timed management of its symptoms significantly improves quality of life [14–16]. IBS can result in anxiety, depression, and even suicide [13]. A higher risk of developing dementia among IBS patients over fifty years old has also been pointed out [17]. Thus, if IBS is triggered or worsen by natural disasters, the fact will be a scientific basis for clinicians to provide early and focused support for disaster victims who have had or are developing IBS symptoms.

Ramosetron, polycarbophil calcium, and mepenzolate bromide are approved solely for the treatment of IBS under the public insurance coverage in Japan; in other words, they cannot be prescribed for other diseases. These three drugs (IBS drugs) cannot be obtained over-the-counter in the market. Thus, a change of the prescription rate for these drugs is considered to reflect the change of apparent incidence, prevalence and severity of IBS among the population. Before a physician prescribes IBS drugs, a series of events must take place: the patient develops bowel symptoms, visits a physician, is diagnosed as IBS, and is judged as a case for applying one of the IBS drugs. Prescription is the final step in this process and indicates the existence of the underlying IBS diagnosis. We thus infer that if the number of prescriptions for IBS drugs among disaster victims is higher than non-victims, it is likely that the disaster causes IBS among the victims.

In terms of the scale of destruction, the 2018 Japan Floods was one of the largest natural disasters in Japan, with only the Great East Japan Earthquake causing more devastation in this century [18]. In the Japan Floods, 263 people died, 8 people went missing, 484 people were injured, 6783 houses completed destroyed, and 11,346 houses partially destroyed as of April 2019 [19]. The floods occurred across western Japan from June 28 to July 8, 2018, causing landslides and river flooding in many areas. Hiroshima, Okayama, and Ehime prefectures were particularly hit hard, with 238 deaths (approximately 90% of total deaths in the nation). The tremendous impacts, included death of family members, interruption of lifelines, and loss of housing forced victims to live in shelters, led to a substantial physical and psychological stress among victims.

This study aims to clarify the impact of the 2018 Japan Floods on the prescription rate of IBS drugs using a national universal health insurance database. This database contains all the prescriptions for IBS drugs and all disaster victims certified by their local governments. We conducted a comparison of the number of prescriptions between victims and non-victims and also between the pre- and post-disaster period. Based on the results, we will show the extent to which the disaster affected the incidence of newly treated IBS patients, how medication for IBS changed among patients treated by IBS drugs, and how long the effect lasted after the disaster.

## Methods

### Study design and data collection

This is a retrospective cohort study using the Japanese National Database of Health Insurance Claims and Specific Health Checkups (NDB) which is one of the administrative claim databases in Japan. We obtained permission to use the data from the Ministry of Health, Labor and Welfare (Permission no. 1223-2). Japan has a universal health insurance system, and the NDB contains all health service claims of everyone who visited any medical institution. The only exceptions comprised less than 2% of the population who receive public income support or were covered with automobile liability insurance or workers’ compensation insurance. Those who were directly affected by the disaster were certified as a victim by the residential local government. To be certified as a victim, a person needed to meet one of the following criteria: (1) the dwelling house was destroyed, damaged, burned down, or flooded; and (2) a family member who had financially supported the victim was killed, injured or missing, had lost a job or suspended business, or was unemployed without income. The certified victims were temporarily exempted from out-of-pocket fees (10–30% of the total cost) for any medical service. This rule applied to the entire population throughout the study period. All victims were tagged with a victim code in the NDB, and the code could be extracted to identify victim status of each subject.

The observation period for this study was from July 2017 to June 2019, extending from 1 year before to 1 year after the disaster. We included study subjects who had visited a medical institution or been hospitalized in Hiroshima, Okayama, and Ehime prefectures in the observational period. Subjects younger than 15 years of age were excluded. Those who had a victim code were assigned to the victim group, and others to the non-victim group. Information on gender, age group (15–19, 20–39, 40–64, 65–79, 80-older), prefectures in which the medical institutes subjects visited, and the total number of prescriptions for any medication 1 year before the disaster were extracted from the NDB. We extracted the name and the number of prescribed IBS drugs (i.e., ramosetron, polycarbophil calcium, and mepenzolate bromide), and duration of the days for which these drugs were prescribed for each subject. We also extracted information on prescriptions for the following drugs (IBS-related drugs) which were listed as symptom controllers for IBS in the Clinical Practice Guidelines for Irritable Bowel Syndrome issued by the Japanese Society of Gastroenterology (JSGE) in 2020 [20]: anticholinergics (thixidium, butyl scopolamine, thimepidium, butropium, and N-methyl scopolamine methyl sulfate), antidiarrheal (loperamide, albumin tannate, berberine chloride hydrate, and bismuth hyponitrate), gastrointestinal motility modifiers (Trimebutine maleate), intestinal secretagogue (lubiprostone and linaclotide), laxative (magnesium, senna, daikon, and sodium picosulfate), and probiotics.

### Drugs for irritable bowel syndrome and definition of users

Many drugs used for IBS are often used for other diseases as well [20]. In this study, we categorized drugs used exclusively for IBS as “IBS drugs” and those that were non-exclusive as “IBS-related drugs”. Regarding IBS drugs, ramostron (5-HT3 receptor antagonists) is approved in Japan only for the diarrhea subtype of IBS, polycarbofil calcium is for diarrhea and constipation subtypes, and mepenzolate bromide is for general symptoms of IBS. Prescriptions of these IBS drugs were evaluated in our main analyses and those of IBS-related drugs were included in sub-analyses. IBS-related drugs were those listed as the first step treatment options for IBS symptoms in the guideline of the JSGE [20]. Probiotics and gastrointestinal motility modifiers are one of the first choices for treatment of IBS and are used regardless of a subtype of IBS (i.e., IBS-constipation, diarrhea, mixed and unclassified), and other IBS-related drugs are used according to a subtype of IBS.

The subjects were categorized into two groups according to their usage status for IBS drugs during the observational period. Patients without any prescription 1 year before the disaster (July 2017–June 2018) were defined as “non-users,” and patients with at least one prescription for the period were defined as “pre-disaster users.”

### Outcomes

To investigate the relationship between the disaster and the development of IBS, we examined how prescriptions for IBS drugs and IBS-related drugs changed before and after the disaster. The prescription rate in our analysis was calculated with the proportion of the occurrence of prescriptions among the target subjects. The rate was calculated by dividing the number of prescriptions for target drugs by the number of target subjects in a month. If more than one type of target drugs was prescribed in a month for a target subject, it was counted as one prescription in that month. Similarly, if target drugs were prescribed more than once in a month for a target subject, it was counted as one prescription in that month. The primary outcome was the monthly occurrence of prescriptions for IBS drugs among all subjects during the observational period. The trend for monthly occurrence rate of prescriptions was compared between the victim and non-victim group. Next, among non-users, we compared the occurrence rate of the prescription 1 year after the disaster between victims and non-victims. If any one of the IBS drugs was prescribed after the disaster, the occurrence of the prescription was counted. We also performed a subgroup analysis among non-users. In the subgroup analysis, the occurrence rate of prescription among non-users was examined in each of the following groups: gender (male or female) and age group (15–64 or 65-).

The secondary outcomes were the monthly occurrence of prescriptions for IBS-related drugs and change of type in the prescribed IBS-related drugs among pre-disaster users during the observational period. The trend for the monthly occurrence rate of prescriptions among pre-disaster users was compared between the victim and non-victim group just as conducted for IBS drugs. We did not distinguish prescriptions for regular use from those for potion. Next, the change of type in IBS-related drugs was also examined using the mean prescription rate 1 year before and after the disaster.

### Statistical analysis

We described the baseline characteristic of participants in this study. The χ^2^ test was used to compare binary and categorical variables between the victim and non-victim group. The continuous variables for non-normal distribution were summarized with median and interquartile range, and were compared between the groups with the Wilcoxson rank sum test.

We evaluated the occurrence of prescriptions for target drugs using two different levels of analysis: a monthly-level analysis and an individual-level analysis. The monthly-level analysis was conducted to evaluate temporal changes of occurrence rate for prescriptions among all subjects and pre-disaster users, while the individual analysis was conducted to evaluate the risk for occurrence of prescription in order to estimate the effect of the disaster on non-users. First, as the main analysis, the monthly occurrence of prescriptions for IBS drugs among all subjects and that of prescriptions for IBS-related drugs among pre-disaster users were examined in the victim group and non-victim group, respectively. The monthly trends of prescriptions during the observational period were compared between the two groups. A controlled interrupted time series design was used to evaluate the disaster-derived change in the occurrence of prescriptions in each group. To estimate the impact of the disaster, we divided two segments: pre-disaster and post-disaster. Then, we compared level and trend between the victim and the non-victim group. The level is the value of the prescription rate at the beginning of a given time interval (i.e., the y-intercept for the first segment and the next segment, which is immediately after the disaster). The trend is the rate of change in prescriptions for IBS drugs (i.e., the slope) during each segment. We used a liner regression model to handle autocorrelation which is the generalized least-squares method. This model included three interaction terms: between victim status and period indicator, between victim status and post-disaster period indicator, and between victim status, post-disaster period indicator and period indicator. The lag period for prescription changes was the month of the disaster (July 2018) because we assumed that the disaster immediately impacted prescriptions for IBS or IBS-related drugs.

Second, as an individual-level analysis, we evaluated the occurrence of prescriptions for IBS drugs among non-users after the disaster. In the analysis, we estimated the risk of new onset of IBS or exacerbation of existing IBS. We used a multivariable logistic regression model, and reported the risk as the odds ratio of the victim against non-victim group. This regression model was adjusted for gender and age group, and the adjusted odds ratio were reported with 95% confidence interval (CI). In addition, subgroup analysis among non-users was performed after a statistical test for interaction. We performed a multivariable logistic regression with an interaction term between gender and victim status, and that between age group and victim status in order to assess the heterogeneity of the impact of the disaster among non-users. P for an interaction value of < 0.05 was considered statistically significant. In subgroup analysis as well, a multivariable logistic regression was used to assess the occurrence of prescriptions for IBS drugs in each group of non-users. We similarly reported the effect as the odds ratio with a 95% CI adjusted for gender or age group.

Analyses were performed using STATA/MP-Parallel Edition version 16.1 (Stata Crop 2019) and 2-sided P values of < 0.05 were considered statistically significant.

### Ethical considerations

The requirement for informed consent was waived because NDB data is anonymous. We obtained study approval from the Ethics Committee of Epidemiological Research, Hiroshima University (Permission no. E-1688).

## Results

The number of subjects included in this study was 5,287,888 out of the 6,176,299 registered in the NDB because subjects younger than 15 years of age were excluded. 32,499 people (0.61%) were categorized into the victim group. Table 1 shows baseline characteristics before the disaster. 50.5% of victims and 34.2% of non-victims were over 65 years old, and the proportion of women was slightly higher in the victim group. The largest number of victims in the study subjects were in Okayama prefecture, accounting for 46.5% of all victims (Additional file 1: Supplementary Table 1). Table 1 also shows non-users accounted for 99.1% of victims as compared with 99.4% of non-victims. Pre-disaster users also accounted for 0.88% of victims, which was higher than that of non-victims, suggesting that the prevalence of patients with IBS was higher in the victim than non-victim group even before the disaster. Victims among non-users were more likely to receive prescriptions before the disaster than non-victims, while the number was not a significant difference between victims and non-victims among pre-disaster users. Among pre-disaster users, the prescription rate of polycarbophil calcium was lower, and that of mepenzolate bromide was higher in the victim than in the non-victim group. There was no significant difference between the two groups in the prescribed days of IBS drugs before the disaster.

**Table 1:** Baseline characteristics before the disaster

	Victims	Non-victims	P value
All participants n	32,499	5,255,389	
Age group			
15–19 n, (%)	1264 (3.9)	286,495 (5.5)	< 0.001
20–39 n, (%)	4654 (14.3)	1,302,881 (24.8)	
40–64 n, (%)	10,185 (31.3)	1,870,036 (35.6)	
65–79 n, (%)	11,237 (34.6)	1,183,566 (22.5)	
80- n, (%)	5159 (15.9)	612,411 (11.7)	
Male n, (%)	14,176 (43.6)	2,449,648 (46.6)	< 0.001
Total number of prescriptions Median (IQR)	7 (1–13)	3 (0–11)	< 0.001
Non-users^*^ n, (%)	32,214 (99.1)	5,222,326 (99.4)	< 0.001
Age group			
15–19 n, (%)	1252 (3.9)	284,480 (5.5)	< 0.001
20–39 n, (%)	4613 (14.3)	1,295,913 (24.8)	
40–64 n, (%)	10,086 (31.3)	1,859,500 (35.6)	
65–79 n, (%)	11,156 (34.6)	1,175,467 (22.5)	
80- n, (%)	5107 (15.9)	606,966 (11.6)	
Male n, (%)	14,058 (43.6)	2,433,409 (46.6)	< 0.001
Total number of prescriptions Median (IQR)	7 (1–13)	3 (0–11)	< 0.001
Pre-disaster users^†^ n, (%)	285 (0.88)	33,063 (0.63)	< 0.001
Age group			
15–19 n, (%)	12 (4.2)	2015 (6.1)	0.03
20–39 n, (%)	41 (14.4)	6968 (21.1)	
40–64 n, (%)	99 (34.7)	10,536 (31.9)	
65–79 n, (%)	81 (28.4)	8099 (24.5)	
80- n, (%)	52 (18.3)	5445 (16.5)	
Male n, (%)	118 (41.4)	16,239 (49.1)	0.01
Total number of prescriptions Median (IQR)	14 (8–21)	13 (6–21)	0.13
Agents for IBS			
Ramosetron	62 (21.8)	7182 (21.7)	< 0.001
Polycarbophil calcium	129 (45.3)	17,661 (53.4)	
Mepenzolate bromide	79 (27.7)	5674 (17.2)	
Any concurrent uses	15 (5.3)	2546 (7.7)	
Prescription days^‡^			
0–100 n, (%)	188 (66)	21,148 (64)	0.21
101–200 n, (%)	16 (5.6)	2828 (8.6)	
201- n, (%)	81 (28.4)	9087 (27.5)	

*IBS* irritable bowel syndrome

*Those who had no history of IBS drugs use during the period of 1 year before the disaster

^†^Those who were prescribed at least once for IBS drugs before the disaster

^‡^The number of days prescribed for IBS drugs during 1 year before the disaster

Table 2 shows the change of the type in IBS-related drugs among pre-disaster users. Probiotics were the most commonly prescribed in both groups before and after the disaster. Some groups of drugs were prescribed disproportionately more often in victims than in non-victims after the disaster; this is particularly the case for laxatives among all drug types.

**Table 2:** Change of prescription for types of IBS-related drugs among pre-disaster users

	Pre-disaster		Post-disaster		Difference between pre- and post-disaster
	Victims (%)	Non-victims (%)	Victims (%)	Non-victims (%)	(Change of victims) − (change of non-victims) (%)
Anticholinergics	0.61	0.77	0.61	0.61	0.11
Gastrointestinal motility modifier	0.47	1.66	0.15	1.23	0.11
Intestinal secretagogue	0.38	0.53	0.23	0.56	− 0.18
Antidiarrheal	2.02	1.68	2.19	1.45	0.4
Probiotics	10.38	9.09	8.54	7.02	0.23
Laxatives	9.59	6.60	11.26	6.27	2
Any concurrent use	8.25	6.80	6.29	5.36	− 0.52
Total	31.70	27.12	29.27	22.51	2.18

The mean prescription rate of IBS-related drugs was compared 1 year before and after the disaster

Difference between pre- and post-disaster was calculated with the difference between change of victims and change of non-victims

*IBS* irritable bowel syndrome

**Fig. 1:**
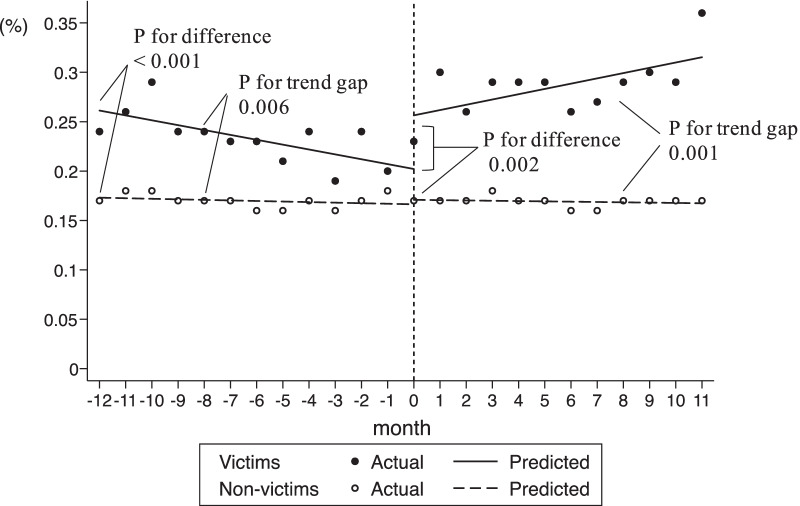
Trends for IBS drug prescriptions among all subjects before and after the disaster. *IBS* irritable bowel syndrome

Figure 1 shows the trend for IBS drug prescriptions among victims and non-victims before and after the disaster. The pre-disaster mean level difference between the victim and non-victim group was significant (0.09%; 95% CI 0.07–0.11; P < 0.001), and the difference in the mean baseline slope was also significant (− 0.005; 95% CI − 0.008 to − 0.001; P = 0.006). In the first month after the disaster (month 0), there appeared to be a significant increase in the prescription rate in the victim group: 0.05% (95% CI 0.02–0.09; P = 0.002). This was followed by a significant increase in the monthly trend for prescription rate (relative to the post-disaster trend for non-victim group): 0.01% per month (95% CI 0.004–0.015; P = 0.001) (Additional file 1: Supplementary Table 2).

**Fig. 2:**
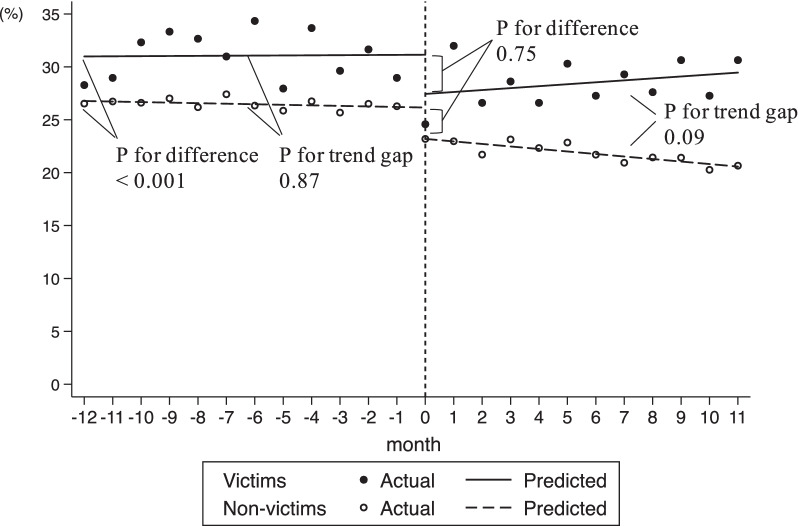
Trends for prescriptions of IBS-related drugs among pre-disaster users before and after the disaster. *IBS* irritable bowel syndrome

Figure 2 shows the trend for prescriptions of IBS-related drugs among pre-disaster users. The pre-disaster mean level difference between the victim group and non-victim group was significant (4.48%; 95% CI 2.21–6.76; P < 0.001), but the difference in the mean baseline slope was not significant (0.02; 95% CI − 0.28 to 0.33; P = 0.87). In the first month of the disaster (month 0), there appeared to be no significant change in the prescription rate at − 0.39% (95% CI − 2.91 to 2.12; P = 0.75), followed by a monthly trend without a significant change in the prescription rate at 0.37% per month (95% CI − 0.06 to 0.79; P = 0.09), relative to the post-disaster trend for the non-victim group (Additional file 1: Supplementary Table 3). The occurrence of prescriptions for IBS-related drugs among victims gradually increased after the disaster, which was in contrast to the flat trend among non-victims.

Among non-users, the occurrence of a prescription for IBS drugs after the disaster was 0.71% for victims and was significantly higher than that for non-victims (0.35%, adjusted odds ratio 2.05; 95% CI 1.81–2.32) (Table 3). In subgroup analysis, a test for interaction terms was performed, and the interaction terms between gender and victim status was significant, while that between age group and victim status was not significant. After the disaster, the occurrence of prescriptions for IBS drugs was significantly higher in women (P for interaction = 0.01), and the heterogeneity among non-users was not found in terms of whether they were the elderly or not (P for interaction = 0.73).

**Table 3:** Occurrence of Prescriptions for IBS drugs After the Disaster Among Non-users

	Victims	Non-victims	Crude OR (95% CI)	Adjusted OR (95% CI)	PI
	n (%)	n (%)			
Non-uses (n = 5,254,540)	241 (0.75)	19,129 (0.37)	2.05 (1.8–2.33)	2.08 (1.84–2.37) ^*^	
Subgroup					
Gender					
Male (n = 2,447,467)	86 (0.81)	9009 (0.37)	1.66 (1.34–2.05)	1.65 (1.34–2.05) ^†^	0.01^§^
Female (n = 2,807,073)	155 (0.85)	10,120 (0.36)	2.36 (2.01–2.77)	2.44 (2.08–2.86) ^†^	–
Age group					
< 65 (n = 3,455,844)	122 (0.76)	12,868 (0.37)	2.05 (1.71–2.46)	2.04 (1.71–2.45)^‡^	0.73^¶^
$\ge$ 65 (n = 1,798,696)	119 (0.73)	6261 (0.35)	2.09 (1.74–2.51)	2.09 (1.74–2.5)^‡^	–

IBS: irritable bowel syndrome; OR: odds ratio; CI: confidence interval; PI: P value for interaction

*OR was evaluated with a logistic regression analysis adjusted for gender and age group

^†^OR was evaluated with a logistic regression analysis adjusted for age group

^‡^OR was evaluated with a logistic regression analysis adjusted for gender

^§^PI between gender (male versus female) and victim status

^¶^PI between age group (< 65 vs $\ge$ 65) and victim status

## Discussion

In this study, we have shown that disaster victims were more likely to be prescribed IBS drugs after the disaster than non-victims. Particularly, among those who had no history of using IBS drugs before the disaster, victims were two-fold more likely to be prescribed IBS drugs than non-victims after the disaster. In other words, the new incidence of IBS supposedly increased, or the number of patients with untreated IBS who required drugs after the disaster increased among victims. Among pre-disaster IBS users, the trend for prescriptions of IBS-related drugs increased among victims after the disaster while it decreased among non-victims. Prescriptions of laxatives, in particular, increased after the disaster suggesting the increased incidence of constipation among victims with IBS.

This is the first study that demonstrated the impact of a large-scale natural disaster on prescriptions for IBS drugs. Another study investigated the incidence of IBS using the Rome III criteria, in middle-school students affected by the Wenchuan Earthquake in China. The incidence of IBS in the earthquake-affected area was reported to be the same as that in the non-affected area [21]. This earthquake study had several limitations: it was conducted as late as three years after the disaster, the sample size was small, there was no distinction between individuals affected directly by the disaster and those non-affected (i.e., the study was vulnerable to “ecological fallacy” in epidemiological terms). In order to overcome these limitations, we conducted this study using a large and reliable database that can separate victims from non-victims among the study population, which in turn has revealed a very different result from that of the previous study.

We assume three reasons for the increased incidence of IBS or the exacerbation of non-treated IBS. First, the disaster negatively impacted mental health among victims. IBS is often described as ‘brain-gut disorder’ because many patients with IBS also have psychiatric disorders and psychotropic drugs are effective for symptoms of IBS [22]. This functional relationship between the brain and gut is an important part of the pathophysiology of IBS [23]. When patients with IBS feel mentally stressed, gastrointestinal symptoms are aggravated [20]. In natural disasters, various mental stresses, due to loss of family or houses, directly affect victims, and the high level of the stress causes depression, insomnia and PTSD among the victims [1]. Subsequently, mentally stressed victims come to require IBS drugs for their bowel symptoms more often than non-victims. Second, the incidence of post-infectious IBS increases among victims because of acute infectious diarrhea caused by the disasters. Deterioration of sanitary conditions due to disasters can lead to an increase in the incidence of infectious diarrhea [6, 7]. The pathophysiology of IBS involves a change of gut microbiota, increased mucosal permeability, and mucosal inflammation; these are strongly related with post-infectious IBS [10]. In addition, it has been pointed out that mental stress, which is also part of the usual sequela of disasters, is a risk for developing post-infectious IBS [12]. Third, it is possible that IBS patients who received psychotherapy cannot continue the treatment after disasters because of reduced accessibility to professional psychotherapists. Psychotherapy is highly effective for symptom relief among IBS patients [24]. The interruption of the therapy potentially causes a worsening of IBS symptoms among victims.

The change in the prescription rate for IBS-related drugs among pre-disaster users may be caused by some food-related factors. Patients with IBS tend to form a habit of evading food that causes symptoms, and some of them even go further to undertake diet therapy such as antigen-eliminating diets based on IgG antibodies [25] and low FODMAP (fermentable oligosaccharides, disaccharides, monosaccharides and polyols) diets [26]. During a disaster, it is highly likely that they are unable to continue these diet patterns. For example, when living in a shelter, they are provided with ready-made or preserved foods, which would usually be avoided in their pre-disaster lives. These emergency foods can exacerbate symptoms of IBS. Constipation, which is caused by changes in food contents and reduced water consumption, would be particularly prevalent in such an emergency situation [3]. The finding in this study that laxatives are used more frequently than any other IBS-related drugs support this hypothesis.

It should be noted that, even before the disaster, victims were prescribed IBS drugs more often than non-victims. A reason for this would be that residents of flood-prone areas (e.g., riverbanks, lowlands, and mountain slopes), which usually are at a lower land price, are likely to be in a lower socioeconomic status. People with a low socioeconomic status are, in general, known to be at a higher risk for psychiatric disorders [27]. Since patients with psychiatric disorders are more likely to have comorbid IBS than those with normal mental health, people of a lower socioeconomic status would receive more IBS drug prescriptions. Thus, as shown in the results of this study, the disaster affected population with a higher IBS prevalence, which leads to an even further widening of IBS prevalence gap between victims and non-victims. In contrast, there may be accessibility bias between the victim and non-victim group. Victims more often received prescriptions for any medications before the disaster than non-victims, which indicates that they visited medical facilities more frequently. This means victims might be a group who had a better accessibility to medical facilities, and such a group of people would access facilities even more after the disaster. When interpreting our results, the escalation of pre-disaster high access among victims should be taken into account.

The frequency and intensity of natural disasters have increased in recent years, and thus physicians increasingly need to be familiar with disaster-related diseases. It is known that uncontrolled symptoms of IBS lead to decreased quality of life for patients with IBS [28]. Although symptoms of IBS may disappear spontaneously during the natural course of IBS [29, 30], early detection of new onset or exacerbation of IBS after disasters leads to better treatment and better quality of life for patients. Physicians should recognize that the JSGE guidelines recommend not to leave symptoms untreated and not to interrupt treatment [20]. Therefore, even in evacuation shelters, it is important to arrange space for victims to maintain privacy and reduce mental stress. In addition, we should consider to providing IBS patients with intestine-friendly and clean diets in order to prevent a worsening of IBS symptoms and acute infectious diarrhea. These measures should be included in policies to prepare for major disasters.

This study has some limitations. First, the diagnosis and the reasons for prescription are unknown because NDB data do not include such information. However, IBS drugs are not available over-the-counter, so people without a definite diagnosis by a physician cannot obtain IBS drugs. In other words, we can identify all patients with IBS who received treatment with IBS drugs. Second, ramosteron might be used for chemotherapy-induced nausea and vomiting or postoperative nausea and vomiting. Although it is used for this purpose in other countries, such use is not approved by Japanese public medical insurance. However, while such use of ramostrone may in fact exist in Japan, we did not exclude prescriptions for this drug according to this use. Third, the diagnostic criteria of IBS (e.g., the Rome IV criteria [31]) might have not been applied to all of our subjects taking IBS drugs. Thus, it is unclear whether the treated patients truly had criteria-met IBS. Fourth, all people affected by the disaster as well as the whole population who lived in the observed prefectures were not necessarily identified. In the NDB, only affected people who applied to the local government for victim certification and visited a medical institution were tagged with a victim code. Thus, we cannot identify affected people who did not apply or visit a medical institution. Furthermore, based on government statistics [32], the population of persons aged 15 and older of Hiroshima, Okayama, and Ehime prefecture as of October 2017 was estimated to be 2,461,000, 1,665,000, and 1,200,000, respectively, which added up to 5,326,000 people. The NDB used in this study identified 5,287,888 subjects, although these subjects included those who died and those who relocated during the observational period. By simple calculation, the number of subjects in this study covered approximately 99% (5,287,888/5,326,000) of the estimated population of the three prefectures, but this proportion might be too high. In one report [33], 26.5% of Japanese households had not visited any medical institution for two months, and in another report [34], 11.5% of households had not visited them for a year. According to these reports, although it is impossible to measure exactly the proportion of people who did not visit any medical institution during the observational period, we can estimate that the proportion was a few percent because the observational period was two years in this study, which was longer than that in the cited reports. In addition, even though there were such people, they would have been distributed in both the victim and non-victim groups in an unbiased manner. Thus, the impact of not being able to count the number of those who did not visit a medical institution would be negligible. Fifth, not all drugs used for IBS were analyzed in this study. Anxiolytics, anti-depressants, and herbal medicines (*Kampo* agents) are also used for this purpose in Japan. These drugs, however, are more often used for other diseases than IBS, and therefore we considered it inappropriate to include them in our analyses.

Finally, it is necessary to consider the possibility that the number of prescriptions of IBS drugs increased among victims because they did not have to pay for the medication themselves. The price elasticity of outpatient care in Japan’s health insurance system has been reported to be − 0.125 to − 0.076 [35]. The co-payment rate in the insurance system is generally 10% for people aged 75 and above, 20% for those aged 70–74, and 30% for those under 70. In this study, the mean co-payment rate for the victim group, based on age distribution, is 23.9%. When the co-payment was totally exempted, the increase in the amount of care demand was calculated as at 1.88–2.99%. Thus, the increase in demand for medical care due to the co-payment exemption would be minimal, and does not explain the substantial increase in prescriptions for IBS drugs.

## Conclusions

The 2018 Japan Floods increased the number of prescriptions for IBS drugs among victims. The results suggest that natural disasters are a risk factor for IBS incidence. It is important for physicians who provide care for IBS patients to understand this risk and start comprehensive care including drug treatment in the early stages of a disaster.

## Supplementary Information

**Additional file 1.** **Supplementary Table 1.** Demographic characteristics of study subjects. **Supplementary Table 2.** Estimates of change in occurrence of prescriptions for IBS drugs among all subjects. **Supplementary Table 3.** Estimates of change in occurrence of prescriptions for IBS-related drugs among pre-disaster users.

## Data Availability

The Japanese National Database of Health Insurance Claims and Specific Health Checkups (NDB) is owned by The Ministry of Health, Labor and Welfare (MHLW). This data of this research was obtained from the NDB which was permitted to use by the MHLW. The data used in this research was only available to the authors who were authorized by the MHLW, and accessing the data was strictly restricted. Requests for access to a full de-identified data of the study by qualified investigators can be addressed to the corresponding author, but it is necessary to obtain the permission to use the data from the MHLW.

## Abbreviations

IBS: Irritable bowel syndrome
PTSD: Post-traumatic stress disorder
NDB: Japanese National Database of Health Insurance Claims and Specific Health Checkups
JSGE: Japanese Society of Gastroenterology
CI: Confidence interval
FODMAP: Fermentable oligosaccharides, disaccharides, monosaccharides and polyols
