# Supplementary material for: Increased prescriptions for irritable bowel syndrome after the 2018 Japan Floods: a longitudinal analysis based on the Japanese National Database of Health Insurance Claims and Specific Health Checkups
Source: BMC Gastroenterol. 2022 May 26;22:263. doi: 10.1186/s12876-022-02342-6 (PMC9137058; doi:10.1186/s12876-022-02342-6)
Supplement: Supplementary file 1 — Additional file 1. Supplementary Table 1. Demographic characteristics of study subjects. Supplementary Table 2. Estimates of change in occurrence of prescriptions for IBS drugs among all subjects. Supplementary Table 3. Estimates of change in occurrence of prescriptions for IBS-related drugs among pre-disaster users. [file 12876_2022_2342_MOESM1_ESM.pdf]

Supplementary Table 1 Demographic Characteristics of Study Subjects

	Victims (n=32,499)	Non-victims (n=5,255,389)	P value
Prefectures			
Hiroshima n, (%)	8,755 (26.9)	2,192,580 (41.7)	<0.001*
Okayama n, (%)	15,111 (46.5)	1,462,068 (27.8)	
Ehime n, (%)	6,206 (19.1)	1,073,717 (20.4)	
Missing or other n, (%)	2,427 (7.47)	527,024 (10.0)	

We compared prefectures in which study subjects visited any medical institution before and after the disaster between the victim and non-victim group.

* P value was estimated with the $\chi 2$ test.

Supplementary Table 2 Estimates of Change in Occurrence of Prescriptions for IBS Drugs Among All Subjects

Measure of Interest	Coefficient	Standard error	95% Confidence interval	P value
Pre-disaster trend: non-victims	-0.001	0.001	-0.002 - 0.001	0.279
Pre-disaster mean level difference: victims versus non-victims	0.090	0.010	0.069 - 0.110	< 0.001
Pre-disaster difference in the mean slope: victims versus non-victims	-0.005	0.002	-0.008 - -0.001	0.006
Post-disaster trend: non-victims	0.007	0.006	-0.005 - 0.018	0.271
Difference pre- and post-disaster: non-victims	0.000	0.001	-0.001 - 0.002	0.696
Difference in the first month of the disaster: victims versus non-victims	0.053	0.016	0.020 - 0.086	0.002
Post-disaster difference in the mean slope: victims versus non-victims	0.010	0.003	0.004 - 0.015	0.001

IBS: irritable bowel syndrome

This was evaluated by a linear regression model with a controlled time series analysis.

Supplementary Table 3 Estimates of Change in Occurrence of Prescriptions for IBS-related Drugs Among Pre-disaster Users

Variable	Coefficient	Standard error	95% Confidence interval	P value
Pre-disaster trend: non-victims	-0.056	0.016	-0.088 - -0.023	0.001
Pre-disaster mean level difference: victims versus non-victims	4.484	1.124	2.211 - 6.756	< 0.001
Pre-disaster difference in the mean slope: victims versus non-victims	0.025	0.150	-0.278 - 0.327	0.869
Post-disaster trend: non-victims	-2.932	0.281	-3.5 - -2.365	< 0.001
Difference pre- and post-disaster: non-victims	-0.184	0.031	-0.246 - -0.122	< 0.001
Difference in the first month of the disaster: victims versus non-victims	-0.392	1.244	-2.906 - 2.122	0.754
Post-disaster difference in the mean slope: victims versus non-victims	0.368	0.210	-0.055 - 0.792	0.087

IBS: irritable bowel syndrome

This was evaluated by a linear regression model with a controlled time series analysis.
